# Necessity of the sleep–wake cycle for synaptic homeostasis: system-level analysis of plasticity in the corticothalamic system

**DOI:** 10.1098/rsos.171952

**Published:** 2018-10-10

**Authors:** S. Assadzadeh, P. A. Robinson

**Affiliations:** 1 School of Physics, The University of Sydney, New South Wales 2006, Australia; 2 Center for Integrative Brain Function, The University of Sydney, New South Wales 2006, Australia

**Keywords:** neural field theory, neuroplasticity, sleep homeostasis, corticothalamic system

## Abstract

Neural field theory is used to study the system-level effects of plasticity in the corticothalamic system, where arousal states are represented parametrically by the connection strengths of the system, among other physiologically based parameters. It is found that the plasticity dynamics have no fixed points or closed cycles in the parameter space of the connection strengths, but parameter subregions exist where flows have opposite signs. Remarkably, these subregions coincide with previously identified regions that correspond to wake and slow-wave sleep, thus demonstrating state dependence of the sign of synaptic modification. We then show that a closed cycle in the parameter space is possible when the plasticity dynamics are driven by the ascending arousal system, which cycles the brain between sleep and wake to complete a closed loop that includes arcs through the opposite-flow subregions. Thus, it is concluded that both wake and sleep are necessary, and together are able to stabilize connection weights in the brain over the daily cycle, thereby providing quantitative realization of the synaptic homeostasis hypothesis.

## 1. Introduction

Sleep has been argued to induce the reorganization of neuronal structure, leading to reinforcement of learning and memory consolidation, but the mechanisms are poorly understood [1–4]. A leading proposal is the synaptic homeostasis hypothesis (SHH) [5]. It postulates that because wakefulness is accompanied by synaptic strengthening in a large fraction of cortical circuits [5], the effects of sleep must be to return the synaptic strengths to a baseline level, in order to prevent runaway synaptic saturation [5]. In the SHH picture, stable levels of synaptic weights are achieved across repeated cycles of net long-term potentiation (LTP) or strengthening and long-term depression (LTD) or weakening, during wake and sleep, respectively [5]. However, the question of why sleep is necessary to fulfil this function remains unanswered and a quantitative explanation is lacking.

According to the simplest version of the SHH hypothesis, memory consolidation in sleep requires synapses to be downscaled without changing the relative strengths of the excitatory and inhibitory synapses [5], but the physiological details of this scenario have not been explored. Experimental evidence suggests that plasticity during wakefulness results in an increase in cortical excitability (strength of the response of neurons to a given stimulation), but there is still dispute as to whether cortical excitatory potentiation [6,7], inhibitory depression [8,9] or a combination of both causes the wakefulness-induced increase of net excitability in the cortex.

Most theoretical modelling of plasticity in the brain has been conducted at the individual neuron level. But it is computationally intractable to study long-term dynamics of large structures of the brain realistically by tracking spike-based interactions of individual neurons. The human cerebral cortex alone consists of tens of billions of neurons, each on average receiving inputs from approximately 4000 others [10,11]. Given that a typical neuron fires at a rate of approximately 10 spikes s^−1^, on average approximately 40 000 presynaptic spikes will drive approximately 10 postsynaptic ones [10,11]. The number of effective presynaptic inputs is actually lower because each neuron will only integrate input spikes within a 10–20 ms time window before firing. Even so, 400–800 presynaptic spikes contribute to the generation of a postsynaptic spike [12]. Thus, the commonly assumed picture of each postsynaptic spike being generated by one or a few presynaptic ones is not accurate, particularly when populations of neurons are involved [11,13]. Multineuron effects must also be incorporated, particularly network oscillations involved in brain rhythms, which correlate large-scale activity [14–16]. There is also the issue of stability. To avoid excessively high or low firing rates, the total excitatory drive to a neuron within a network must be tightly regulated, which is difficult to do if synapses are modified independently. Rate or activity-based Hebbian rules have been developed to tackle this problem [11,17]; in these models, it is assumed that the rate of pre- and postsynaptic firing measured over some time period, determines the sign and magnitude of synaptic plasticity [10]. Rate-based plasticity has been shown to capture many features of single-neuron spike-timing-dependent plasticity (STDP) if the analysis preserves temporal correlations [10,11,18].

Physiologically based neural field theory (NFT) is well suited to the analysis of large-scale brain dynamics, where firing rates are averaged over neuronal populations. NFT has been successfully used in the past to quantitatively predict brain electrical activity, including EEG time series and spectra [15,19,20], coherence and correlations [16], evoked response potentials (ERPs) [15,19], seizure dynamics [15] and plasticity [11,21], among other phenomena, and has also enabled automated arousal state estimation [15,22].

Plasticity in NFT can be written as a generalized learning rule that preserves the relative timings of input and output activity, allowing the representation of both correlation-dependent plasticity (CDP) and STDP dynamics [11,21]. In this formulation, the average plasticity responses depend upon temporal correlations between presynaptic inputs and postsynaptic responses. This enables one to derive the expected correlation, given the transfer functions that map inputs to responses, and then to explore network-level effects [11,21]. Spectral analysis of plasticity applied to a population of excitatory neurons with external input and feedback has revealed the strong role of system-level resonances in plasticity, including the reversal of the plasticity sign at different frequencies (transitions from LTD to LTP and vice versa) [11]. These results provide a starting point for a quantitative description of synaptic homeostasis via sleep and wake, as they demonstrate that the sign of plasticity can change between arousal states, as the shape of the power spectrum changes [11]. However, exploration of this last point requires study of the full corticothalamic (CT) system.

Recently, NFT was used to investigate plasticity in a cortical model with both excitatory and inhibitory populations [21]. Using physiologically based estimates of the plasticity parameters, it was found that synaptic strengths are unstable, thus necessitating other stabilizing mechanisms such as the saturation of synaptic strengths, or modified plasticity rules, in order to avoid the divergence of synaptic strengths [21]. Thus, the problem of maintaining large-scale stability in a plastic brain remains open.

We approach the synaptic sleep-homeostasis problem via analysis methods from systems biology, neuroscience and physics. We first extend previous works and study plasticity in the CT system, and discuss the role of system-level plasticity in synaptic homeostasis. We show that the plastic CT system is unstable, with no fixed points. We then demonstrate that if we allow some of the CT connections to have a different plasticity window, plasticity can have opposite signs (LTP versus LTD) in sleep and wake. We then show that it is possible to achieve synaptic homeostasis by accounting for the sleep–wake cycle, which moves the brain parametrically between states of opposite flow (sign of synaptic modification), thereby stabilizing the system.

## 2. Theory

In this section, we briefly review NFT, the CT system and its stability, the parametrization of the model into arousal states and the plasticity formulation used [11,15,16,20–25].

### 2.1. Neural field model

The brain contains multiple populations of neurons, which are distinguished by a subscript *a* that designates both the structure in which a given population lies, and the type of neurotransmitter it expresses. Using a continuum approximation, their properties are averaged over scales of approximately 0.1 mm, resulting in mean-field quantities. The relationships between these quantities are captured in the NFT equations.

The mean soma potential *V*_*a*_(***r***, *t*), measured relative to resting, is generated when synaptic inputs from afferent neurons *b* are temporally low-pass filtered and smeared out in time as a result of receptor dynamics, passage through the dendrites, and soma responses of neurons *a*; it approximately obeys the differential equation [19]

2.1 $$D_{ab}V_{a}(r,t)=\sum_{b}\nu_{ab}\phi_{b}(r,t-\tau_{ab})$$

and

2.2 $$D_{\alpha \beta }=\frac{1}{\alpha_{a}\beta_{a}}\frac{{\text{d}}^{2}}{\text{d}t^{2}}+\left(\frac{1}{\alpha_{a}}+\frac{1}{\beta_{a}}\right)\frac{\text{d}}{\text{d}t}+1,$$

where ***r*** = (*x*, *y*) denotes the spatial coordinates and 1/*β*_*ab*_ and 1/*α*_*ab*_ are characteristic rise and decay times of the potential due to an impulse at a dendritic synapse. The right of equation (2.1) describes the influence of the pulse rates *ϕ*_*b*_ arriving at population *a* from neuronal populations *b*, in general delayed by a time *τ*_*ab*_ when there are discrete anatomical separations between different structures. The quantity *ν*_*ab*_ = *N*_*ab*_*s*_*ab*_, where *s*_*ab*_ is the time-integrated response in neurons of type *a* to a unit signal from neurons of type *b* (which is negative for inhibitory synapses), and *N*_*ab*_ is the mean number of synapses [19,20,24,25]. Thus, *ν*_*ab*_ denotes the average connection strength of synapses to population *a* from population *b*. Changes in *s*_*ab*_ are modelled by plasticity dynamics and we explore this later in the section.

Action potentials are produced at the axonal hillock when the soma potential *V*_*a*_ exceeds a threshold, at a rate that rises steeply with *V*_*a*_ before levelling off. In a population, this dependence is smeared out by differences in individual neurons and their environments to yield the population-average response function [15,25]

2.3 $$Q_{a}(r,t)=S[V_{a}(r,t)]$$

and

2.4 $$S[V_{a}(r,t)]=\frac{Q_{\max}}{1+\exp\{-[V_{a}(r,t)-\theta ]/\sigma^{\prime }\}},$$

where *S* is a sigmoid function that limits *Q* to *Q*_max_ as *V*_*a*_ increases to ∞, and we assume a common mean neural firing threshold *θ* relative to resting, with $\sigma^{\prime }\pi /\sqrt{3}$ being its standard deviation [20,25].

Each neuronal population *a* itself produces a field *ϕ*_*a*_ of pulses that travels to other neuronal populations at a velocity *v*_*a*_ through axons with a characteristic range *r*_*a*_. This activity spreads out and dissipates if not regenerated. To a good approximation, this type of propagation obeys the damped wave equation [15,25]

2.5 $$D_{a}\phi_{a}(r,t)=Q_{a}(r,t)$$

and

2.6 $$D_{a}=\frac{1}{\gamma_{a}^{2}}\frac{\partial^{2}}{\partial t^{2}}+\frac{2}{\gamma_{a}}\frac{\partial }{w\partial t}+1-r_{a}^{2}\nabla^{2},$$

where the damping coefficient is *γ*_*a*_ = *v*_*a*_/*r*_*a*_. Equations (2.1)–(2.6) form a closed nonlinear set, which can be solved numerically, or examined analytically in the linear limit [19,20,24,25].

### 2.2. Steady states and dynamics

Spatially uniform steady states are obtained by setting all space and time derivatives in equations (2.1)–(2.6) to zero. The stable steady-state solutions are interpreted as representing the baseline of normal activity, which yields firing rates in accord with experiment [12,15,20], with time-dependent brain activity arising from the perturbations. Previous work has successfully modelled normal brain states as lying in the linear regime [19,25–27], allowing observable quantities such as transfer functions, spectra, and correlation and coherence functions to be expressed analytically. Thus, we linearize equation (2.3) by considering only first-order perturbations and write

2.7 $$Q_{a}(r,t)-Q_{a}^{\left(0\right)}\approx \rho_{a}[V_{a}(r,t)-V_{a}^{\left(0\right)}]$$

and

2.8 $$\rho_{a}={\left.\frac{\text{d}S(V_{a})}{\text{d}V_{a}}\right|}_{V_{a}=V_{a}^{\left(0\right)}}.$$

From here on, we consider perturbations from steady states so equation (2.7) becomes

2.9 $$Q_{a}^{\left(1\right)}(r,t)=\rho_{a}V_{a}^{\left(1\right)}(r,t),$$

where $Q_{a}^{\left(1\right)}$ and $V_{a}^{\left(1\right)}$ are perturbations [15,19,20].

The Fourier transform of equations (2.1)–(2.8) result in the following spectral representation of travelling activity, which is particularly useful for exploring network dynamics [12]:

2.10 $$\phi_{a}^{\left(1\right)}(k,\omega )=\sum_{b}L(\omega )\;{\text{e}}^{i\omega \tau_{ab}}G_{ab}\Gamma_{a}^{\left(0\right)}(k,\omega )\phi_{b}^{\left(1\right)}(k,\omega ),$$

2.11 $$L(\omega )={\left(1-\frac{\text{i}\omega }{\alpha }\right)}^{-1}{\left(1-\frac{\text{i}\omega }{\beta }\right)}^{-1}$$

2.12 $$\;\mathrm{and}\;\Gamma_{a}^{\left(0\right)}={\left[{\left(1-\frac{\text{i}\omega }{\gamma_{a}}\right)}^{2}+\;k^{2}r_{a}^{2}\right]}^{-1},$$

where ***k*** and *ω* are the wavevector (with magnitude *k* = 2*π*/λ where λ is the wavelength) and angular frequency (*ω* = 2*πf* where *f* is the frequency in Hz), respectively and $\Gamma_{a}^{\left(0\right)}$ and *L*(*ω*) are derived from the Fourier transforms of the differential operators defined in equations (2.6) and (2.2), respectively. Here, the gains are defined by *G*_*ab*_ = *ρ*_*a*_*ν*_*ab*_ = *ρ*_*a*_*N*_*ab*_*s*_*ab*_ and denote the differential response in neurons *a* per unit input from neurons *b*.

### 2.3. Plasticity dynamics

Each postsynaptic spike in a neuron actually results from multiple incoming spikes and interacts with the effects of all presynaptic spikes to produce plastic changes at their afferent synapses, so a sum over their effects is necessary to determine the overall effect [10,21] and it is not possible, even in principle, to determine which presynaptic spike leads to the generation of a particular postsynaptic one, because all the presynaptic spikes are integrated at the soma. In any event, at the spatial and temporal scales of interest here, we keep the essential dynamics by considering the instantaneous NFT axonal pulse rates *ϕ*_*b*_, and postsynaptic firing rates *Q*_*a*_. In doing so, we preserve the average temporal correlations between pre- and postsynaptic activity that drive plasticity.

The rate of synaptic modification is denoted by d*s*_*ab*_/d*τ*, where *τ* represents time on the plasticity timescale. Following Hebb's rule, d*s*_*ab*_/d*τ* is proportional to the integral (as *ϕ*_*b*_ and *Q*_*a*_ are continuous) of the correlations of input and output activity, within a temporal plasticity window *H*_*ab*_(Δ*τ*), where Δ*τ* = *t*_*a*_ − *t*_*b*_ is the time delay between the post- and presynaptic activity at times *t*_*a*_ and *t*_*b*_, respectively. Thus, we have [11,28]

2.13 $$\frac{\text{d}s_{ab}(r,\tau )}{\text{d}\tau }=B\int_{-\infty }^{\infty }Q_{a}^{\left(1\right)}(r,t+\tau_{ab})H_{ab}(\Delta \tau )\phi_{b}^{\left(1\right)}(r,t)\;\text{d}\Delta \tau ,$$

where *t* relates to timescales much shorter than that of plasticity. The infinite bounds of the integral are not a problem because the rapid decrease of the window function *H*_*ab*_(Δ*τ*) effectively cuts off the integral at a few tens of milliseconds. However, we also know that synaptic modification occurs on a much slower timescale than the neuronal dynamics [11,18], implying that only the temporal average of the right-hand side of equation (2.13) drives net plasticity dynamics. Hence, we average equation (2.13) over a timescale *T*, where *T* is much longer than the width of the plasticity window. From here on, the superscripts (0) and (1) are omitted for brevity so that the quantities *Q*_*a*_, *ϕ*_*a*_ and *V*_*a*_ denote perturbations from resting state values. Equation (2.13) can now be written as [11]

2.14 $$\frac{\text{d}\langle s_{ab}\rangle }{\text{d}\tau }=B\int H_{ab}^{*}(\omega )\Gamma_{b}^{*}Q_{a}(r,\omega )Q_{b}^{*}(r,\omega )\frac{\text{d}\omega }{2\pi },$$

where $\Gamma_{b}={\left[{\left(1-\text{i}\omega /\gamma_{b}\right)}^{2}+k^{2}r_{b}^{2}\right]}^{-1}$ is the Fourier transform of the operator *D*_*a*_ in equation (2.6), and the term *Q*_*a*_(***r***, *ω*) Q*_b_ (***r***, *ω*) is the cross spectrum between *Q*_*a*_ and *Q*_*b*_ at ***r***.

Experimental results have shown that the shape of plasticity window depends on the type of synapse [10], and can even vary under different conditions for the same synapse [17]; however, it is well known that the effect of plasticity is reduced at longer spike time intervals. A widely used approximation assumes that the plasticity window decays exponentially [10,29], so that in evaluating equation (2.13) we use

2.15 $$H_{ab}(\Delta \tau )=A^{+}\exp\left(-\frac{\Delta \tau }{t_{p}}\right),\;\Delta \tau >0,$$

2.16 $$=A^{-}\exp\left(\frac{\Delta \tau }{t_{p}}\right),\;\Delta \tau \leq 0,$$

where *t*_p_ is the plasticity timescale and the *A*^±^ are constants. This general form includes all possible combinations of signs of CDP and STDP.

Fourier transformation of equations (2.15) and (2.16) gives

2.17 $$H_{ab}(\omega )=\frac{H_{0}+\text{i}\omega t_{\;p}H_{1}}{1+{\left(\omega t_{\;p}\right)}^{2}},$$

with *H*_0_ = (*A*^+^ + *A*^−^) *t*_p_ and *H*_1_ = (*A*^+^ − *A*^−^) *t*_p_, where *H*_1_ = 0 and *H*_0_ = 0 correspond to CDP and STDP, respectively. Equation (2.17) implies that CDP tends to be more sensitive to the lowest *ω* contributions of the other factors in the integrand in equation (2.14), while STDP is preferentially driven by activity at *ω* ∼ 1/*t*_p_.

### 2.4. Corticothalamic model

**Figure 1.**
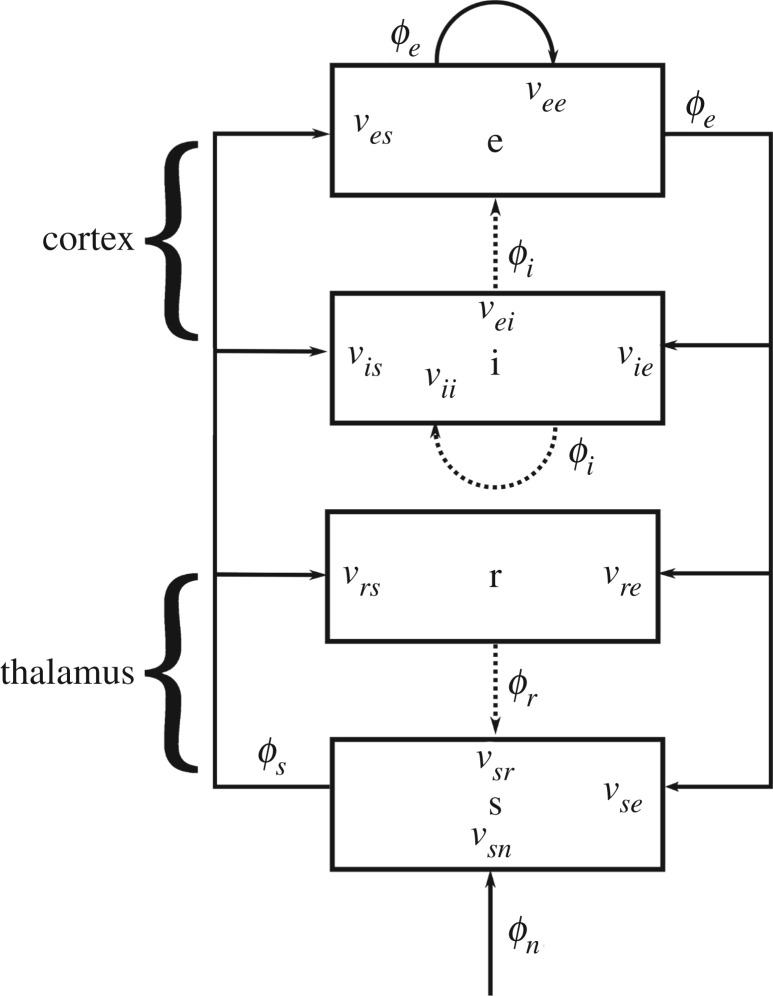
CT system diagram. Connections are represented by arrows that show connectivity between the cortical excitatory (*e*) and inhibitory (*i*), and thalamic relay (*s*) and reticular (*r*) populations. The inhibitory connections are shown as dotted arrows, which denotes that they inhibit activity in the populations to which they project [19].

The CT model used here describes the interactions of four populations: cortical excitatory (*e*), cortical inhibitory (*i*), thalamic reticular (*r*) and thalamic relay (*s*) [24]. The connectivity between these populations is shown in figure 1. The system is driven by the external stimulus *ϕ*_*n*_ to the thalamic relay nuclei, which project activity to the thalamic reticular nucleus and the cortex. The long-range cortical axons connect distant regions of the cortex to excitatory populations or inhibitory populations, forming a cortical feedback loop, while some of the excitatory axons connect the cortex to the thalamic populations *r* and *s*. The populations *i* and *s* are inhibitory, meaning that they inhibit activity in the populations to which their axons project. In this case, *r* and *i* suppress activity in the relay nuclei (*s*) and the cortex, respectively.

Equation (2.10) for the CT system gives four simultaneous equations describing each of the pulse rates *ϕ*_e_, *ϕ*_i_, *ϕ*_r_ and *ϕ*_s_ in terms of incoming activity from other populations and the external stimulus *ϕ*_*n*_. Synapses projecting outwards from a given population have the same chance of being connected to an inhibitory or excitatory cortical population (random connectivity) [24]. Using this simplification (i.e. *ν*_*ib*_ = *ν*_*eb*_), we can eliminate *ϕ*_i_, *ϕ*_r_ and *ϕ*_s_ to obtain the transfer function to *ϕ*_e_. In particular [15,20,25],

2.18 $$\frac{\phi_{e}(k,\omega )}{\phi_{n}(k,\omega )}=\frac{G_{esn}\;\text{exp}(\text{i}\omega t_{0}/2)}{\left(1-G_{srs}L^{2})(1-G_{ei}L)(k^{2}r_{e}^{2}+q^{2}r_{e}^{2}\right)},$$

and

2.19 $$q^{2}r_{e}^{2}={\left(1-\frac{\text{i}\omega }{\gamma_{e}}\right)}^{2}-\frac{1}{1-G_{ei}L}\left\{LG_{ee}+\frac{[L^{2}G_{ese}+L^{3}G_{esre}]\;{\text{e}}^{i\omega t_{0}}}{1-L^{2}G_{srs}}\right\},$$

where *G*_*esn*_ = *G*_*es*_*G*_*sn*_ and the quantities *G*_*ese*_ = *G*_*es*_*G*_*se*_, *G*_*esre*_ = *G*_*es*_*G*_*sr*_*G*_*re*_ and *G*_*srs*_ = *G*_*sr*_*G*_*rs*_ correspond to the overall gains for the excitatory CT, inhibitory CT and intra-CT loops, respectively. The EEG power spectrum *P*(*ω*) can then be calculated by integration of |*ϕ*_*e*_(**k**, *ω*)|^2^ over all spatial modes *k*, which can be evaluated analytically for some forms of the input stimulus *ϕ*_*n*_, including white noise [20,27]. In this case, a spatio-temporal white noise input gives |*ϕ*_*n*_(**k**, *ω*)|^2^ = const., and the frequency spectrum is [20]

2.20 $$P(\omega )=\frac{\left\langle \phi_{n}^{2}\right\rangle }{4\pi r_{e}^{2}}{\left|\frac{G_{esn}L^{2}}{\left(1-G_{srs}L^{2})(1-G_{ei}L\right)}\right|}^{2}\left|\frac{\text{Arg}\;(q^{2}r_{e}^{2})}{\text{Im}\;(q^{2}r_{e}^{2})}\right|,$$

where $\left\langle \phi_{n}^{2}\right\rangle$ is the mean-square noise level, Arg denotes the complex argument, and Im is the imaginary part. This power spectrum has been shown to match closely with EEG spectral data from normal subjects, making white noise a good approximation for the stimuli that drive spontaneous activity [20]. The characteristic shape of the EEG power spectrum then depends on the wave number *k*, the temporal parameters *α*, *β*, *γ* and *t*_0_ and the gains *G*_*ee*_, *G*_*ei*_, *G*_*ese*_, *G*_*esre*_ and *G*_*srs*_.

### 2.5. Stable zone

The space of stable brain states (i.e. the set of gains where the system is linearly stable) can be represented in a three-dimensional space, which is easier to visualize than the five-dimensional set of gains in the full model [20,22,27]. The reduced parameters enable simple expressions for the locations of certain stability boundaries, from which strengths of resonances, damping and general features of the power spectrum can be determined. These three dimensions are defined by

2.21 $$X=\frac{G_{ee}}{1-G_{ei}},$$

2.22 $$Y=\frac{G_{ese}+G_{esre}}{\left(1-G_{srs})(1-G_{ei}\right)}$$

**Figure 2.**
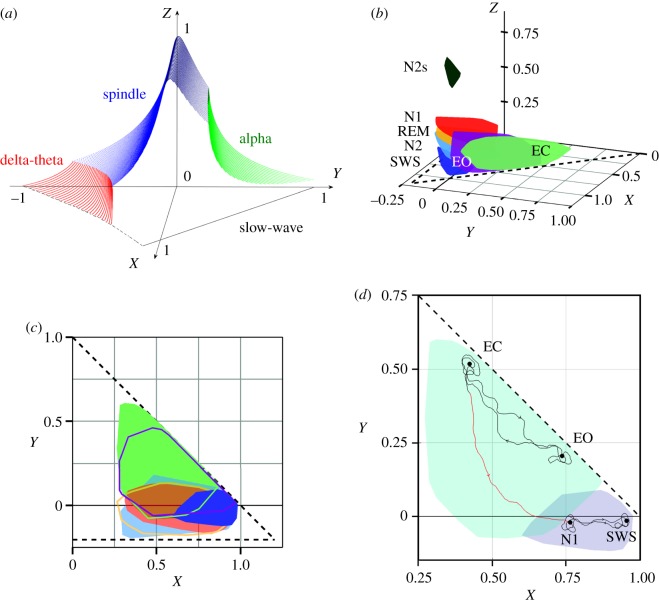
Structure of parameter space and representation of arousal stages in *XYZ* space. (*a*) Stability zone in the reduced CT model, for the parameters listed in table 1 [27]. Colours indicate the dominant resonances close to the instability boundary, as labelled, with the front right-hand face (*X* + *Y* = 1 plane) left unshaded. (*b*) Model parameter regions corresponding to wake states and the traditional sleep stages; EO, eyes open; EC, eyes closed; SWS, slow wave sleep; N2s, sleep spindles; N1 and N2 are sleep stage 1 and 2, respectively. For each stage, the convex hull of the corresponding brain states is shown in the three-dimensional representation of the model parameters. (*c*) Projections of the arousal stages onto the *XY*-plane, using the same colours as frame (*b*). The dashed line *X* + *Y* = 1 represents the zero-frequency instability boundary, while the horizontal dashed line corresponds to the region where *P*(*ω*) ∝ *ω*^−3^: the steep low-frequency spectrum seen in deep sleep [22,27]. (*d*) Illustration of typical trajectories in state space, with black curves representing the changes in parameters during a particular state, and red lines corresponding to the wake–sleep transition.

2.23 $$\;\mathrm{and}\;\bar{Z}=-G_{srs}=\frac{{\left(\alpha +\beta \right)}^{2}}{\alpha \beta }Z,$$

where the auxiliary quantity $\bar{Z}$ is introduced for brevity. The quantities *X*, *Y* and *Z* parametrize corticocortical, CT and intrathalamic loop strengths, respectively. The stable zone (SZ) of neural activity can now be visualized in *XYZ* space as shown in figure 2*a*. The colours indicate the dominant frequency bands close to the boundary. SZ consists of all points in parameter space below this surface, and bounded by the planes *Z* = 0 and *X* = 0, because of physiological constraints. The gains *G*_*ei*_ and *G*_*sr*_ are negative because they correspond to inhibitory connections between their respective populations, while the remaining gains are positive. The values of *X* and *Z* are therefore constrained to positive values, whereas *Y* can be positive or negative depending on the balance between excitatory and inhibitory CT feedback. The plane *X* + *Y* = 1 corresponds to a saddle–node bifurcation [15], where states beyond it have been shown to characterize pathological brain activity, including epileptic seizures [15,27]. Healthy brain states, such as normal arousal states, must lie within the stability zone [22,24].

### 2.6. Arousal stages and states

The brain's state evolves continuously in XYZ space, within the SZ shown in figure 2*a*. The changes in the physiological quantities in the brain reflect the continuously evolving arousal levels and the corresponding dynamics. Recent work by Abeysuriya and Robinson [23] successfully tracked these physiological quantities in real time by fitting the model's power spectrum to that of EEG spectra from healthy subjects, showing that brain states evolve continuously, even when transitioning between wake and sleep.

Despite the fact that the brain's state evolves continuously, arousal states are traditionally artificially discretized into a small set of ‘stages’: wake, REM (rapid eye movement) sleep and three stages of non-REM sleep (see American Association of Sleep Medicine (AASM) classification) [30]. Stage 1 sleep or N1 corresponds to light sleep and is usually short in duration; stage 2 sleep (N2) is a deeper stage of sleep marked by K-complexes (each typically having a large negative peak in the EEG, followed by a positive peak, similar to an evoked response) and sleep spindles (short bursts of activity around 12–14 Hz), labelled N2s; and slow wave sleep (SWS or N3), which corresponds to deep sleep in which K-complexes and sleep spindles are sometimes present. Rapid eye movement (REM) sleep occurs during dreaming and is often the final stage of a standard sleep cycle [30].

Fits of the model to observed EEG power spectra can be carried out to infer the model parameters, and hence the location of its state in *XYZ* [22]. The phenomenologically discrete arousal stages can thus be replaced by a continuous representation of brain states in *XYZ* space that reflects the continuous dynamics of the brain's CT system, rather than sudden artefactual transitions between coarsely discretized stages [22].

The mapping of the various AASM arousal stages into *XYZ* space is shown in figure 2*b*, with the projections onto the *XY* plane shown in figure 2*c* [15,22]. Note that each stage maps to a volume of space, not a point, because of inter- and intra-subject variability within the state. In general, wake states are associated with smaller values of *X* (cortical) and larger positive values of *Y* (CT), than sleep states [22]. The larger values of *Y* indicate a strong positive feedback between the cortex and the thalamus, which is a characteristic of a strong alpha peak arising from the CT loop. The alpha peak is especially enhanced near the alpha stability boundary at which the CT feedback would cause an instability [15,22].

Smaller *X* values (weak cortical feedback) reduce the amount of delta power present, which is observed as flattening of the spectrum at low frequencies during waking states. During sleep, there is a weaker CT interaction, suppressing the alpha peak. During slow wave sleep (SWS) in particular, the delta power is large, with a steep low-frequency slope, indicating a strong intracortical coupling (*X*) and near-criticality (*X* + *Y* ≈ 1), and placing these states at larger *X* and smaller *Y* than wake states. The values of *Z* correspond to the intrathalamic feedback and can be used to distinguish sleep spindles and lighter sleep stages (N1 and N2) from SWS [22].

Figure 2*d* shows an example of a typical trajectory in state space, where the trajectories within wake and sleep states are indicated by the black curves, while the red curves represent the transition between wake and sleep. Note that we use the AASM stages to indicate approximate parameter regions, to illustrate these stages in *XYZ* space (tables 1 and 2).

**Table 1.** Nominal estimates of parameter values for the CT model, found in previous work [20].

quantity	estimate	unit
*α*	83	s^−1^
*β*	769	s^−1^
*t*_0_	0.085	s
*Q*_max_	340	s^−1^
*γ*_*e*_	116	s^−1^
*γ*_*i*_	1500	s^−1^
*r*_*e*_	0.086	m
*σ*′	0.006	V
*θ*	0.0204	V
*N*_*ee*_	10^4^	—
*N*_*ei*_	1600	—
*N*_*es*_	800	—
*N*_*se*_	1100	—
*N*_*sr*_	550	—
*N*_*sn*_	450	—
*N*_*re*_	2700	—
*N*_*rs*_	700	—

**Table 2.** Typical parameters for representative examples of each arousal stage [22]. EO, eyes open; EC, eyes closed; S1, S2 for sleep stage 1 and 2, respectively, and slow wave sleep (SWS).

quantity	EO	EC	REM	N1	N2	SWS	spindles	units
*G*_*ee*_	10.5	2.1	5.9	7.5	16.9	19.5	18.5	—
−*G*_*ei*_	13.2	4.1	6.6	8.3	17.9	19.7	19.0	—
*G*_*es*_	1.2	0.77	0.21	0.31	3.9	5.3	2.6	—
*G*_*se*_	5.8	7.8	0.66	1.7	0.07	0.22	0.73	—
−*G*_*sr*_	2.8	3.3	0.28	0.40	0.14	0.22	0.26	—
*G*_*sn*_	14.2	8.1	0.68	3.9	2.4	1.7	2.8	—
*G*_*re*_	0.85	0.66	2.1	7.5	5.0	1.9	4.7	—
*G*_*rs*_	0.25	0.20	4.6	4.4	8.3	1.4	16.9	—
*X*	0.74	0.41	0.77	0.80	0.89	0.94	0.93	—
*Y*	0.17	0.51	0.00	−0.01	−0.06	−0.04	−0.01	—
*Z*	0.06	0.06	0.20	0.28	0.18	0.05	0.70	—

## 3. Results and discussion

### 3.1. Plasticity drive

Plasticity dynamics are governed by equation (2.14), where the rate of change of the connection strengths depends on the spectral features of the system at any given moment. Thus, we expect system-level effects, such as resonances to play a strong role in the rate of synaptic modification. To demonstrate this, we evaluate equation (2.14) at the parameter values representative of the traditional arousal stages given in table 2, which correspond to seven points in *XYZ* space.

**Figure 3.**
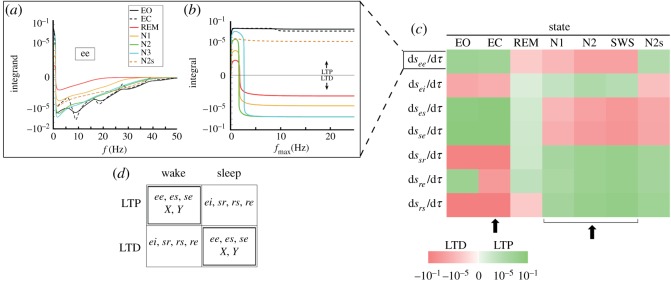
State dependence of plasticity in the CT connections. (*a*) Plasticity integrand for the *ee* connection (excitatory cortico-cortical plasticity), with the corresponding integral in (*b*). Integrands and integrals in equation (2.14) evaluated at seven different arousal states (given in table 2), with plasticity parameters given in table 3. The values on the vertical axis are scaled with an inverse hyperbolic sine function, so that the scaling is linear close to zero and logarithmic elsewhere, while preserving the signs. All tick marks are separated by one order of magnitude. The labels LTP and LTD are used to identify regions where connections are strengthened or weakened (in magnitude), respectively. (*c*) Summary of the sign and magnitude of plasticity in all the CT connections, at the representative arousal states. The black arrows indicate the states where the sign of plasticity is opposite, in all the connections. (*d*) LTP and LTD of CT connections in wake and sleep.

The integrand Γ*_b_(*ω*) *Q*_*a*_(*ω*) H*_ab_(*ω*) Q*_b_(*ω*) for the *ee* connection is shown in figure 3*a*, which illustrates the contribution of the different frequency components to the total synaptic modification rate d〈*s*_*ab*_〉/d*τ*. The integrand shows strong resonances at *f* = *f*_*α*_, 2*f*_*α*_, 3*f*_*α*_, … , where *f*_*α*_ ≃ (*t*_0_ + 2/*α* + 2/*β*) ^−1^, which coincides with the alpha peak and its harmonics. Hence, there is a significant contribution to the plasticity integral at these resonances. Plasticity at frequencies above 50 Hz is negligible because of the low-pass cut-off set by |*L*(*ω*)|. Thus, it is sufficient to evaluate the integrals only up to an upper bound of *f*_max_ = 50 Hz; however, the integral is already very close to its limit by *f*_max_ ≈ 10 Hz in figure 3*b*. Note that frequencies up to 5 Hz provide a large contribution to plasticity because |*L*(*ω*)| peaks at *f* = 0. The other main feature of plasticity is the sign change at *f* ∼ 1 Hz, where the plasticity integrand crosses the horizontal axis. This means that the contributions to d〈*s*_*ee*_〉/d*τ* are positive for f≲1 Hz and negative for f≥1 Hz, with particular enhancement in the 1–3 Hz range during sleep. The fact that the plasticity can change from LTP to LTD is particularly important for applications such as transcranial magnetic stimulation (TMS), where the sign of synaptic modification depends on the stimulus frequency [11], but this point is not pursued further here. Finally, it can be seen that the rate of synaptic modification is state dependent, such that *s*_*ee*_ undergoes LTP during wake, because of the net positive contribution from all frequencies up to *f*_max_; and similarly, LTD takes place during sleep (except in the transient sleep spindles where the plasticity sign is similar to wake states).

The algebraic sign of plasticity during the eyes closed wake state (EC) is positive for the *ee*, *ei*, *es*, *sr* and *se* connections, negative for the *rs* and *re* connections, and vice versa for all connections during sleep (N1, N2 and SWS). Later in §3.3, we find regions within wake that include both EC and EO, where the sign of plasticity is opposite to those of regions within sleep. For the excitatory connections, d*s*_*ab*_/d*τ* > 0 implies an increase of synaptic strengths |*s*_*ab*_| (or LTP), as expected under the SHH. However, because *s*_*ab*_ is negative for inhibitory connections, d*s*_*ab*_/d*τ* > 0 results in the weakening of the connections; i.e. d|*s*_*ab*_|/d*τ* < 0 and is therefore interpreted as LTD. Thus, as illustrated in figure 3*c*, wakefulness is accompanied by LTP of the *ee*, *es* and *se* connections, LTD of the *ei*, *sr*, *rs* and *re* connections, and vice versa for all the connections during sleep. Note that during wakefulness, this leads to increased excitation in the cortex; because both the reduction of inhibition (LTD of *ei*) and increase in excitatory activity as a result of LTP of the *ee* connections, contribute to this effect.

### 3.2. Dynamics in state space: no fixed points

The plasticity rates evaluated at the illustrative points considered in the previous section lie in a broader continuum of arousal states. To check stability in the state space, we compute d*s*_*ab*_/d*τ* throughout this space. The boundaries where the plasticity sign changes; i.e. where d*s*_*ab*_/d*τ* = 0, define the nullclines of the system. Each of these is the set of points where one of the strengths *s*_*ab*_ ceases to change. Any fixed points must thus correspond to a simultaneous intersection of all nullclines, where all *s*_*ab*_ become stationary.

No fixed points were found for different values and combinations of the plasticity window parameters listed in table 3, and in general, the high-dimensional nature of the gain space implies that it is very unlikely that the nullclines have a common intersection point. And even if a fixed point existed for a special combination of parameters, it would not be robust to parameter changes. Experimental evidence also suggests that the synaptic strengths in the brain (globally) do not converge to a fixed point, rather, synaptic stability is achieved cyclically, with periods of strengthening and weakening, such as in wakefulness and sleep, as expressed by the SHH [1,2,31]. Hence, the non-existence of robust fixed points seems to imply and explain the necessity for cyclic-driven dynamics to maintain stable states in the long-term (multi-day) timescale.

**Table 3.** Plasticity window parameters and corresponding estimates for which regions of opposite flow exist in the CT state space. In all the windows, *A*^+^ > 0 and *A*^−^ < 0.

parameter	estimate	unit
*A*_*ee*_^+^/*A*_*ee*_^−^	−1.01	—
*A*_*ei*_^+^/*A*_*ei*_^−^	−1.01	—
*A*_*es*_^+^/*A*_*es*_^−^	−1.25	—
*A*_*re*_^+^/*A*_*re*_^−^	−1.00	—
*A*_*rs*_^+^/*A*_*rs*_^−^	−0.40	—
*A*_*sr*_^+^/*A*_*sr*_^−^	−1.25	—
*A*_*se*_^+^/*A*_*se*_^−^	−1.25	—
*A*_*sn*_^+^/*A*_*sn*_^−^	−1.25	—
*t*_*p*_	0.01	s

### 3.3. Cyclic CT stability via the ascending arousal system

One possible way to stabilize the connection strengths in the long term is for the system to alternate between regions in parameter space where the plasticity signs are opposite to one another, but this requires an external mechanism to move the system between these regions. An analogous solution can be found in the stability problem of the inverted pendulum, an example of which is a long pole balanced on its base, so it has a natural tendency to tip over. Cyclic stability of the connections in the CT system can be achieved analogously to balancing an inverted pendulum, which involves periodic adjustments to the pivot point at the base of the pole. There, as the pendulum begins to fall in one direction, it can be kept from falling by moving the pivot point in the same direction, whereby the pendulum begins to accelerate in the opposite direction. One solution therefore involves a sinusoidal oscillation of the pivot between two points (the reader can try this by balancing a long ruler on a finger). Similarly, we argue that the brain is able to avoid long-term divergences of connection strengths by spending time in sleep, where the flows are opposite to those of wake, and transitioning between these two states via parameter changes before the synaptic weights deviate too far from their long-term means. This is the essence of the SHH [5], but we examine it in the full CT system.

Typical plasticity parameters that enable regions of opposite flows to exist are listed in table 3, which were also used in producing the plots in figure 3. We find that if the same plasticity window is used for all the CT connections, in addition to the dynamics being unstable (no fixed points), not all connections have regions of opposite flow within connection strength space. Notably, once the windows for the intracortical and *rs* connections are adjusted as in table 3, opposite flow regions exist for all connections, and these overlap in parameter space (i.e. one set of flow regions all overlap, and the corresponding opposite-flow regions also all overlap). The plasticity dynamics are found to be robust to small changes in the window function parameters, and small perturbations are observed to change the size of these opposite flow regions. The plasticity windows themselves are physiologically constrained, with positive $A_{ab}^{+}$ and negative $A_{ab}^{-}$; i.e. LTP and LTD occur, respectively. What is even more remarkable is that, even though no *a priori* constraints are imposed on the locations of the two sets of regions, we find that they lie in exactly the subregions of parameter space that belong to wake and sleep.

**Figure 4.**
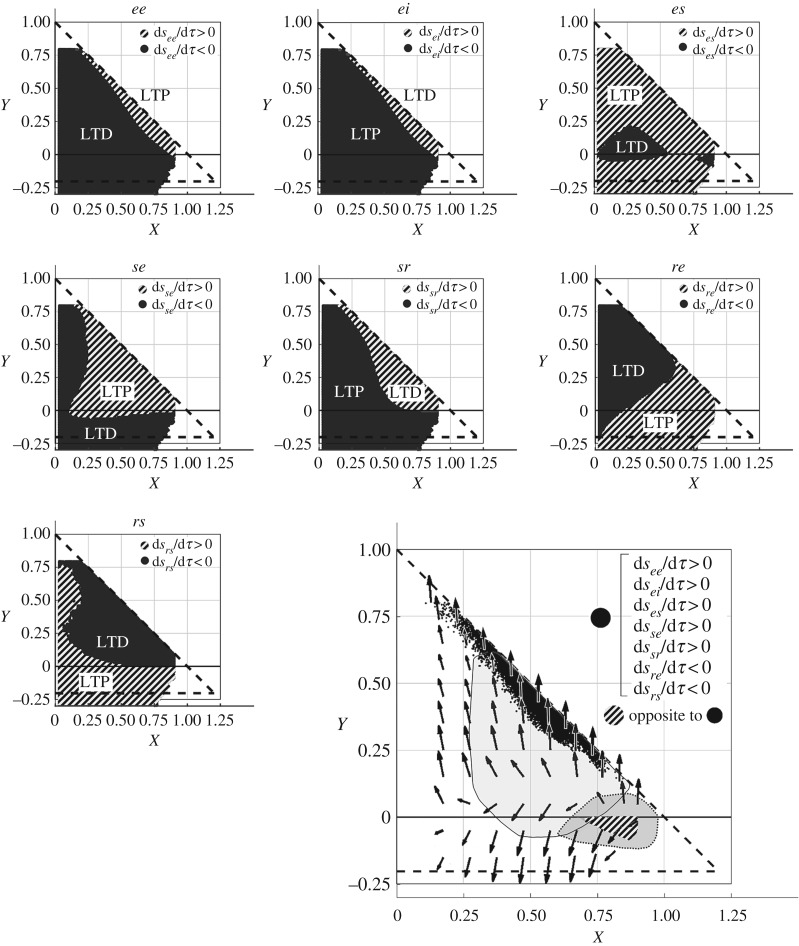
Plasticity flows in arousal state space. Plots in the small panels showing the sign of plasticity (d*s*_*ab*_/d*τ*) at each of the CT connections, at different points in arousal state space (projection onto *XY*). The plasticity window parameters used are listed in table 3. Diagonally shaded regions correspond to d*s*_*ab*_/d*τ* > 0, which represent LTP in the excitatory connections and LTD in the inhibitory connections (*ei* and *sr*). Areas in black correspond to d*s*_*ab*_/d*τ* < 0. The larger panel shows regions of opposite flow, with the vectors (d*X*/d*τ*, d*Y*/d*τ*) represented by the black arrows. The areas enclosed by the thin solid and dotted lines correspond to parameter ranges corresponding to wake and SWS, respectively.

Subregions within wake and sleep (SWS in particular) where the signs of d*s*_*ab*_/d*τ* are opposite are shown in figure 4 in the large panel. The black region shows the set of points where the connections *ee*, *es* and *se* undergo LTP, while *ei*, *sr*, *rs* and *re* are subject to LTD. The plasticity signs of individual connections in state space are portrayed in the small panels. There are parameter regions in the *XY*-plane where LTP and LTD overlap because of the projection of the eight-dimensional *s*_*ab*_ space onto the *XY*-plane. To simplify the visual depiction of these regions, we have uniformly sampled points in the *XY*-plane and averaged the normalized velocities of those points over regions of size 0.01 ×0.01.

**Figure 5.**
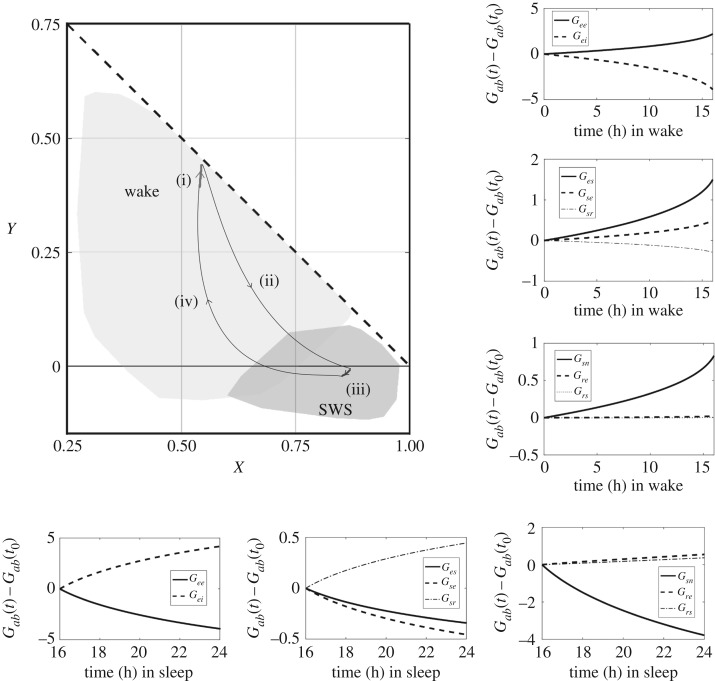
Evolution of gains in a wake–sleep cycle. The larger panel shows the trajectories in *XY* space (plasticity windows listed in table 3). Light grey areas correspond to wake states with dark grey representing SWS. The diagonal dashed line represents the zero-frequency instability boundary. A closed cycle in state space is formed by processes labelled (i)–(iv). Processes (i) and (iii) are driven by plasticity during wake and SWS, respectively. The AAS drives the sleep–wake transition, here approximated by the thin black lines labelled (ii) and (iv). The corresponding time series of the individual gains (difference from onset value at time *t*_0_) during wake and sleep are plotted in the smaller panels. Connections that undergo LTP switch to LTD and vice versa during wake and sleep.

The fact that the ascending arousal system (AAS) pushes the system back and forth between wake and sleep, and thus between regions of opposite flow [32], provides a mechanism to parametrically stabilize the system. The shifts in parameters between wake and sleep are due to the neuromodulatory influences of the AAS [32]. Neurons that cause sleep are located in the ventrolateral pre-optic area of the hypothalamus (VLPO) and they inhibit the arousal system by releasing GABA and galanin neurotransmitters. When the arousal system is active, the VLPO neurons become silent and the subject becomes awake [32]. An example of a typical closed trajectory during the wake–sleep cycle is shown in figure 5.

### 3.4. Evolution of synaptic strengths across sleep and wake

The initial state within the wake subregion (the dark region in the large panel in figure 4) is evolved forward in time according to the plasticity integral in equation (2.14). The trajectories are computed using a second-order numerical Euler predictor-corrector scheme, which updates the connection strengths at successive timesteps, with step size *h* = 10^−4^ s. At each new set of values of the *s*_*ab*_, the system is linearized to obtain the new gains of the system. The initial state within the wake subregion evolves to near its maximum possible limit, close to the instability boundary of *X* + *Y* = 1. The largest change in strength occurs in *ee*, whose connection strength will have increased by approximately 40%. To correspond to a normal 2 : 1 ratio of wake to sleep, the plasticity-driven trajectory within the sleep subregion (the diagonally shaded regions in figure 4) is evaluated for half the duration of the wake trajectory. During the sleep phase, *ee* is found to decrease by approximately 30% (of the now larger than original strength) during sleep, so that synaptic strengths return close to their baseline levels. Typical changes in synaptic strengths over a diurnal cycle have been estimated to be tens of per cent across a single wake–sleep cycle [5], with a study on mice demonstrating that after a 24 h period of single whisker stimulation, the total synaptic density in the corresponding cortical barrel increases by 36% [33]. It is also worth noting that many other possible closed-loop trajectories exist near the illustrative one shown.

In the cortex during wake, |*s*_*ee*_| increases (LTP), while |*s*_*ei*_| decreases (LTD). The net effect is cortical potentiation or increase in the corticocortical loop gain (d*X*_wake_/d*τ* > 0). Wake is also accompanied by net CT potentiation; i.e. d*Y*_wake_/d*τ* > 0. Thus, flows during wake push the system closer to the zero-frequency instability boundary at *X* + *Y* = 1, beyond which pathological dynamics occur [15]. Synaptic plasticity is shown to push the system parameters towards this instability boundary, which may account for the observed increase in the likelihood of seizures due to sleep deprivation [8], a phenomenon that is currently not well understood. The evolution of the corresponding gains during wake and SWS are shown in the smaller panels in figure 5. The initial gains *G*_*ab*_(*t*_0_) are chosen to lie within the opposite flow regions identified in figure 4 and the difference of the gains from the initial value, *G*_*ab*_(*t*) − *G*_*ab*_(*t*_0_), is plotted on the vertical axis, with the horizontal axis representing time.

During sleep, flows in parameter space are reversed with respect to flows in wake, so that d*X*_sleep_/d*τ* < 0, d*Y*_sleep_/d*τ* < 0, which correspond to cortical and CT depression. Without the sleep–wake transition, cortical and CT gains would continue to decrease until the brain reaches coma-like states and eventually all cortical activity would cease because the cortical excitatory synapses are strictly depressed, and inhibitory connections are always potentiated, in this region of parameter space. This is shown in figure 4, in the panels showing regions of LTP and LTD for the *ee* and *ei* connections, respectively. Close to *X* = 0.8 and *Y* = 0 (sleep), *s*_*ee*_ decreases, while d|*s*_*ei*_|/d*τ* > 0. Hence, wake and sleep are both necessary for synaptic homeostasis. The transition back to wake is driven by the AAS, which takes place on a timescale much faster than the plasticity timescale. The trajectory that a brain takes during a sleep to wake (or wake to sleep) transition is not very well known; however, in EEG recordings, it is observed that transition to sleep states from wake begins with the decrease of the low-frequency power (≲5 Hz), followed by the inhibition of the alpha and beta bands; and vice versa for the sleep-to-wake transition [23]. The trajectories representing this effect in *XY*-plane are sketched in figure 5 as thin lines connecting wake and sleep regions. Thus, the combined actions of plasticity and the AAS lead to stability in the brain by forming closed cycles in state space.

## 4. Summary and conclusion

We have used NFT to study plasticity in the CT system, consisting of cortical excitatory and inhibitory, and thalamic reticular and relay populations. The CT system has previously been shown to have regions in the space of synaptic connection strengths that correspond to normal arousal states and we have used plasticity to explore the flows in this space and show that cyclic sleep–wake dynamics are required in order to stabilize long-term average synaptic strengths. This places the SHH on a quantitative footing that incorporates system-level effects. The main results are as follows:

(i) We have shown that system-level effects in the brain, including excitatory and inhibitory influences, and the feedbacks and resonances that arise from interactions of cortical and thalamic populations, play a significant role in global plasticity dynamics.

(ii) Plasticity theory shows that the rate of synaptic modification depends on the input spectra from other populations and external stimuli, and also on the resulting spectral response in each population. Our analysis shows that the total plasticity rate is an integral over all frequencies, and that the sign of the contribution can be different at different frequencies. CT resonances (e.g. at the alpha frequency) can make significant contributions to overall plasticity, but the main contributions are from frequencies below 5 Hz in most cases.

(iii) There are no fixed points for the CT system in synaptic strength space, which means that the brain cannot attain a steady state of synaptic strength if it stays in a single arousal state. Rather, wake states would approach seizure instabilities, and sleep states evolve toward coma-like states (see also point (vi) below).

(iv) Subregions exist in parameter space between which all the flows reverse, with LTP becoming LTD, and vice versa, in all the connections. The existence of these simultaneous (for all connections) opposite-flow subregions requires the plasticity window to be different in some of the connections. In particular, the LTD inducing part of the plasticity window is larger than the LTP one for the *rs* connection, unlike all the other CT windows. The windows in the intracortical connections have a slightly larger LTP inducing part, and the connections involving the relay nuclei (*s*) have a similar window, where the LTD part of the window is 80% of the LTP inducing part, except in the *rs* connection, as mentioned above.

(v) Remarkably, without such a constraint being imposed in advance, the opposite-flow regions in (iv) correspond to the wake and non-REM sleep zones of parameter space. Hence, if the brain is moved periodically between these regions, a closed loop can be formed that leads to long-term stabilization of synaptic strengths, despite these having diurnal variations.

(vi) The ascending arousal system (AAS) is known to move the brain back and forth between wake and sleep, and therefore between the opposite-flow subregions. For the relevant parameters in these regions, it is observed that wakefulness is accompanied by LTP of the *ee*, *es* and *se* connections and LTD of the *ei*, *sr*, *rs* and *re* connections, resulting in net cortical and CT potentiation (*X* and *Y* both increase). Left unchecked, as potentiation continues, the system would eventually become unstable, drifting outside the stable region of state space (outside the SZ), possibly increasing the likelihood of seizures. Similarly, synaptic strengths during sleep alone are unstable, and with prolonged sleep the synaptic modifications eventually suppress cortical activity, and the brain would gradually drift into coma-like states characterized by large cortical and thalamic inhibition. Thus, sleep and wake are both necessary for synaptic homeostasis in the brain, because the cyclic plasticity dynamics stabilize synaptic strengths in the long term.

(vii) The observed reversal of the sign of synaptic modification in the excitatory cortical connections (*ee*) from LTP in wake to LTD in sleep is consistent with Tononi and Cirelli's SHH picture [5]. However, our analysis goes beyond the original SHH, in that we have shown that plastic flows reverse in all the other CT connections. The connections between the cortex and the thalamic relay nucleus (*es*, *se*) are found to undergo LTP during wake, with LTD in SWS. The opposite state-dependent plasticity sign is observed for the connections to and from the reticular nuclei (*re*, *rs*, *sr*), with LTD and LTP in wake and SWS, respectively. The magnitude of the changes inferred is of the same order (tens of per cent) as those that originally helped to motivate the SHH, and we find that the largest ones are in the *ee* connections.

(viii) The above findings are consistent with numerous studies that have found that prolonged wakefulness results in cortical excitatory potentiation [6,7] and inhibitory depression [8,9], consistent with the observed *ee* LTP and *ei* LTD that takes place during wake in our model. It has also been observed that boosting slow oscillations (≲1 Hz) during sleep potentiates memory [34], consistent with our finding that *ee* connections can strengthen if the LTP contributions at ≲1 Hz are enhanced (figure 3*a*).

In summary, we have provided the first quantitative support for the SHH, demonstrated the homeostatic role of sleep and found the plasticity conditions and constraints necessary to achieve the alternation of LTP and LTD during wakefulness and sleep. The system-level plasticity analysis that we used has revealed insights beyond the original SHH, which have enabled us to model and track the evolution of synaptic strengths on scales that involve the cortical and thalamic populations responsible for generating the observed large-scale oscillations in the brain. The changing brain activity and dynamics in the CT system across the wake–sleep cycle is found to be of key significance to synaptic homeostasis, resulting in opposite plasticity flows in all the CT connections in the wake and SWS regions of parameter space. Thus, we can explain more clearly how and why both wake and sleep are necessary to stabilize connection strengths in the brain on multi-day timescales and longer.

## Data accessibility

All relevant data are provided in the main text.
